# Infant Sleep: A Precursor to Adult Sleep?

**DOI:** 10.1371/journal.pbio.0030168

**Published:** 2005-04-19

Sleep is absolutely essential for well-being. Just ask one of the 40 million Americans with sleep disorders who suffer crippling fatigue, impaired judgment, irritability, moodiness, and myriad health problems. Still, its precise function remains unclear. An intriguing role for REM sleep—the stage most closely associated with dreaming—was suggested almost 40 years ago when sleep researchers Howard Roffwarg and William Dement discovered that babies spend far more time in REM sleep than adults—prompting their hypothesis that infant REM sleep plays a role in central nervous system development.

A central element of their hypothesis revolves around the nature of infant sleep and whether the neural mechanisms of infant sleep differ significantly from those of adult sleep. Infant rats, like the offspring of other “altricial” species (born naked, helpless, and blind), spend most of their time in what's now called active sleep, indicated by intermittent muscle twitching and low muscle tone (atonia)—behaviors characteristic of adult REM sleep. At issue is whether infant mechanisms are primitive, undifferentiated, and distinct from adult mechanisms or whether they contain elementary components that are integrated into the developing sleep system.

In a new study, Karl Karlsson, Mark Blumberg, and their colleagues tackle the technical difficulties involved in studying the tiny neonatal brain to investigate the neural activity associated with infant sleep states. The active sleep of week-old rats, they show, bears a striking resemblance to the conventional definitions of adult sleep. What's more, the neural mechanisms underlying the infant sleep state contain the primary components of adult sleep.

In previous studies, Karlsson and Blumberg discovered a brainstem region in the ventromedial medulla, which they called the medullary inhibitory area (MIA), that appears functionally equivalent to the region that generates REM atonia in adults. They also found that the MIA doesn't generate infant sleep on its own but depends on a network that spans both lower brainstem and midbrain regions. In this study, the authors set out to identify the neural structures that project to the MIA and better characterize the network.[Fig pbio-0030168-g001]

**Figure pbio-0030168-g001:**
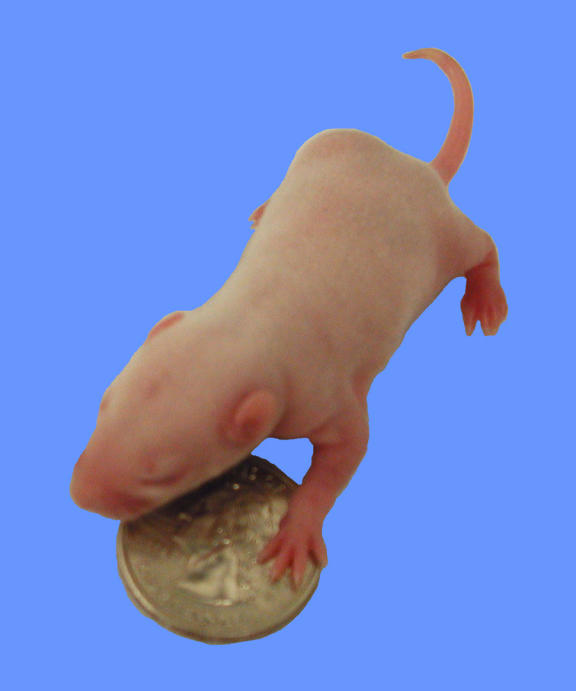
Though naked, helpless, and blind, this week-old rat (pictured with a quarter) already has the fundamental neural components of adult sleep (Photo: Mark Blumberg)

Karlsson et al. first established that there are neurons that connect to the MIA from areas in the medulla and pons. Then, by recording from neurons in these areas, they found neurons that are active only during sleep or wakefulness and that appear to control muscle tone and twitching. Neurons active mostly during atonia—indicating sleep—concentrated in the subcoeruleus (SubLC) region of the pons; those active mostly during wakefulness clustered in an area within the dorsolateral pontine tegmentum (DLPT) in the midbrain. The authors went on to link different sets of neurons with specific behaviors and brain regions. A group of neurons within the DLPT, for example, showed distinct bursts of activity just before muscle twitching. And a subset of SubLC neurons fired at much higher rates when atonia was accompanied by bouts of tail and neck muscle twitching.

Introducing lesions in the SubLC and another pontine nucleus, called the pontis oralis, caused significant changes in muscle tone and twitching. Lesions in the two pontine nuclei reduced periods of atonia but not the number of muscle twitches—in effect decoupling the key components of REM sleep, twitching and atonia. Lesions in the DLPT had the opposite effect: increased atonia and significantly less muscle twitching.

Altogether, the authors argue, these results show that sleep development elaborates on elementary components already in place soon after birth. If the neural mechanisms of infant and adult sleep were entirely different, then sleep might serve different purposes in infancy and adulthood. But the striking parallels outlined in this study suggest a developmental continuity between the two states. They also chart a course for future study that might even test Roffwarg's view that the neonatal brainstem primes the central nervous system for the sensory challenges that lie ahead—and could even be the stuff that dreams are made of.

